# Enhancement in Photoelectrochemical Performance of Optimized Amorphous SnS_2_ Thin Film Fabricated through Atomic Layer Deposition

**DOI:** 10.3390/nano9081083

**Published:** 2019-07-28

**Authors:** Weiguang Hu, Truong Thi Hien, Dojin Kim, Hyo Sik Chang

**Affiliations:** 1 Graduate School of Energy Science and Technology, Chungnam National University, Daejeon 305–764, Korea; 2 Department of Materials Science and Engineering, Chungnam National University, Daejeon 305–764, Korea

**Keywords:** Amorphous SnS_2_ film, Atomic layer deposition, Photoelectrochemical performance, Deposition temperature, Thickness, Photoelectron separation

## Abstract

Two-dimensional (2D) nanomaterials have distinct optical and electrical properties owing to their unique structures. In this study, smooth 2D amorphous tin disulfide (SnS_2_) films were fabricated by atomic layer deposition (ALD), and applied for the first time to photoelectrochemical water splitting. The optimal stable photocurrent density of the 50-nm-thick amorphous SnS_2_ film fabricated at 140 °C was 51.5 µA/cm^2^ at an oxygen evolution reaction (0.8 V vs. saturated calomel electrode (SCE)). This value is better than those of most polycrystalline SnS_2_ films reported in recent years. These results are attributed mainly to adjustable optical band gap in the range of 2.80 to 2.52 eV, precise control of the film thickness at the nanoscale, and the close contact between the prepared SnS_2_ film and substrate. Subsequently, the photoelectron separation mechanisms of the amorphous, monocrystalline, and polycrystalline SnS_2_ films are discussed. Considering above advantages, the ALD amorphous SnS_2_ film can be designed and fabricated according to the application requirements.

## 1. Introduction

One of the effective approaches to overcome the issues related with the increasing energy crisis and environmental pollution is water splitting using the “endless” sunlight to produce and utilize clean energy hydrogen (H_2_) [1,2]. Therefore, it is essential to propose novel cheap and environmentally friendly materials that can efficiently utilize the sunlight. Recently, an increasing number of two-dimensional (2D) materials have been studied, which are suitable for water splitting applications. Their 2D structures and large specific surface areas are beneficial for the carrier transport and interface electrochemical reaction [3,4]. For example, hydrogen gas has been successfully prepared using 2D materials, such as CdS [5], MoS_2_ [6,7], and SnS_2_ [8,9]. Among them, SnS_2_ is an n-type semiconducting material with a layered cadmium iodide-(CdI_2_)-type structure. It can absorb visible light owing to its suitable optical band gap and has a high carrier mobility, which is beneficial for a rapid transmission of photon-generated carriers. In addition, considering its low price, nontoxicity, and good stabilities in neutral and even acid solutions, SnS_2_ is an ideal material for photoelectrochemical (PEC) water splitting [8,9]. However, there are few literatures on PEC water splitting with SnS_2_ as the catalyst. To prepare cost-effective, highly active, and simply manufactured SnS_2_ films is still a challenge.

The properties of SnS_2_ significantly vary with the morphology and preparation method. The reported SnS_2_ morphologies mainly consist of quantum dots [10], nanoparticles [11,12], nanowires [13], flower-like structures [12,14,15,16], and nanosheets (NSs) [8,9,11,12,14]. In addition, SnS_2_ can be combined with other elements and the morphologies can change to nanoplates [17] and nanospheres [18]. Various methods have been used to prepare SnS_2_ crystal structures, including the solvothermal method [8,14,16], hydrothermal method [9,10,12,19], Sn metal sulfuration [13], chemical vapor transport [20,21], chemical vapor deposition [22,23], and atomic layer deposition (ALD) [24,25]. Among them, ALD is a popular film fabrication method, particularly suitable for the fabrication of 2D materials. Firstly, based on sequential self-limiting reactions, ALD provides an excellent conformality and uniformity over large areas. Then, ALD provides an atomic-scale thickness control and tunable film composition, which can control the performance of the film. Moreover, as ALD processes are maintained at modest temperatures (<350 °C), it is easy to obtain an amorphous film [26,27]. In contrast to crystalline films, amorphous films have no cleavage surface. They have better toughnesses and can tightly bind to the substrate in large areas, so some performances may be better than those of crystals [28,29]. Amorphous MoS*_x_* has been used to prepare high-quality photoelectrode for PEC sensing [29] and highly active hydrogen evolution catalysts [30]. However, no PEC applications of amorphous SnS_2_ have been reported.

In this study, ALD was used to prepare smooth 2D amorphous SnS_2_ thin films at low temperatures. Through field-emission scanning electron microscopy (FESEM) and atomic force microscopy (AFM) observations, we confirmed that the amorphous SnS_2_ films were smooth and were in close contact with the substrate. X-ray diffraction (XRD), Raman spectroscopy, X-ray photoelectron spectroscopy (XPS), and high-resolution transmission electron microscopy (HRTEM) analyses showed that amorphous SnS_2_ films were obtained when the preparation temperature was below 140 °C. By optimizing its optical band gap and thickness, the optimal stable photocurrent density (0.8 V vs. saturated calomel electrode (SCE)) of the 50-nm-thick amorphous SnS_2_ film prepared at 140 °C was 51.5 µA/cm^2^, which is better than those of most polycrystalline SnS_2_ films. The equivalent circuit was obtained using electrochemical impedance spectroscopy (EIS). Last, schematic diagrams of photoelectron separation and PEC water splitting are presented. These results indicate that the ALD amorphous SnS_2_ films are promising for PEC water splitting and other applications involving photoelectric transformation.

## 2. Materials and Methods

### 2.1. Tin Disulfide Film Synthesis and Characterization

Tin sulfide (Sn*_x_*S*_y_*) films were deposited by ALD (Lucida^TM^ D, NCD, Daejeon, Korea) on a glass substrate and silicon wafer using a tetrakis (dimethylamino) tin (IV) (TDMASn: 99.99%, Trust Chem, Seoul, Korea) precursor and hydrogen sulfide (H_2_S mixture with nitrogen gas (N_2_), H_2_S: 10.04%) as tin (Sn) and sulfide (S) sources, respectively. N_2_ was employed to carry the precursors and remove excess precursors or reaction products. The flow rate of the N_2_ gas was set to 50 sccm by a mass flow controller to maintain the reaction pressure of 0.38 Torr. The N_2_ purge time between Sn and S pulses was 12.0 s. The H_2_S flow rate was set to 30 sccm. Under a sufficient S supply, various Sn pulse times (0.2, 0.5, 1.0, 1.5, 2.0, 3.0, and 5.0 s) were tested until the reaction reached self-saturation. The optimal S pulse time was obtained with the same approach. The optimized ALD growth cycle was defined by the following sequence; Sn pulse (2.0 s), N_2_ purge (12.0 s), H_2_S pulse (1.0 s), then N_2_ purge (12.0 s), at 140 °C. Subsequently, approximately 50 nm thick Sn*_x_*S*_y_* films were fabricated at deposition temperatures in the range of 60 to 180 °C. Their thicknesses and surface morphologies were observed using ultrahigh-resolution SEM (S-4800, Hitachi, Ltd., Tokyo, Japan). The surface roughness was measured by AFM (Park XE7, Park Systems, Suwon, Korea). The crystal structures and phases of the films were measured by high-resolution XRD (D8DISCOVER, Bruker AXS Inc., Madison, Wisconsin, USA) using Cu K_α_ radiation (1.5406 Å), high-performance Raman spectrometry (LabRAM HR-800, HORIBA Jobin Yvon, Montpellier, France) using a 633-nm laser line, and XPS (MultiLab 2000, Thermo Scientific, Seoul, Korea). The crystal structure and chemical composition was confirmed by HRTEM (JEM-2100F, JEOL USA, Inc., Peabody, MA, USA).

### 2.2. Optical Properties of the Amorphous SnS_2_ Films

In order to analyze the effects of the growth temperature on the optical properties of the amorphous SnS_2_ films, thin films were fabricated at different temperatures (60, 100, and 140 °C) on glass substrates. Their transmittances and absorbances were then measured by UV–Vis spectroscopy (UV-2600, Shimadzu Corporation, Tokyo, Japan) in the wavelength range of 300 to 1400 nm, and their optical band gaps were analyzed.

### 2.3. Photoelectrochemical Performances of the Amorphous SnS_2_ Films

Amorphous SnS_2_ films with different thicknesses (25, 50, 75, and 100 nm) were deposited on fluorine-doped tin dioxide (FTO) transparent conductive glass substrates at different temperatures (60, 100, and 140 °C). Specimens with effective areas of 1 × 1 cm^2^ were then prepared and used as working electrodes. The PEC measurements were performed in a conventional three-electrode system using an electrochemical workstation (AUT84826, Metrohm AG, Herisau, Switzerland) under an AM 1.5G simulated sunlight illumination (100-W Xe arc lamp, model 11002 SunLite^TM^ Solar Simulators, Abet Technologies, Inc., Milford, CT, USA). A platinum (Pt) wire and SCE were used as the counter and reference electrodes, respectively. Linear sweep voltammograms under intermittent illumination and dark conditions were measured with a scanning rate of 5 mV/s in the range of −0.7 to 0.9 V (vs. SCE). The EIS measurements were carried out in the frequency range of 1000 to 0.1 kHz at a bias potential of 0.8 V (vs. SCE) under illumination. In addition, the stability of the photocurrent response was evaluated at 0.8 V (vs. SCE) during 360 s. The measurement of incident photon-to-current conversion efficiency (IPCE) (Model 10500 low cost solar simulator, Abet Technologies, Inc., Milford, CT, USA) was performed in a bias potential of 0 V (vs. reversible hydrogen electrode (RHE)). All of the above analyses were carried out at room temperature.

## 3. Results and Discussion

### 3.1. Tin Disulfide Film Deposition

#### 3.1.1. Atomic layer deposition Model

**Figure 1:**
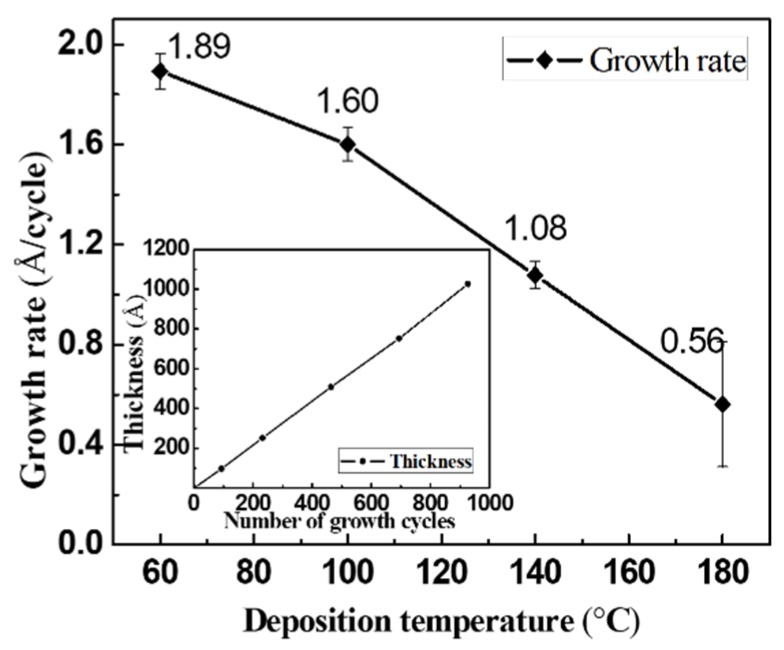
Growth rates of the Sn*_x_*S*_y_* films at deposition temperatures in the range of 60 to 180 °C. The thickness of the Sn*_x_*S*_y_* film linearly increased with the number of growth cycles at 140 °C (inset).

The self-saturation state of the ALD process can be achieved by alternately changing the pulse times of the tin and sulfur sources. The optimal Sn*_x_*S*_y_* film deposition sequence of one ALD cycle was determined to be: Sn pulse (2.0 s), N_2_ purge (12.0 s), H_2_S pulse (1.0 s), then N_2_ purge (12.0 s). Under this deposition condition, the growth rates of the Sn*_x_*S*_y_* films at different deposition temperatures are shown in Figure 1. The growth rate decreased with the increase in the deposition temperature in the range of 60 to 180 °C, which is similar to a previous result for tin sulfides fabricated by ALD using the same Sn source [24]. The inset shows the variation in the film thickness with the number of growth cycles at 140 °C. The thickness was proportional to the number of growth cycles, which indicated that the Sn*_x_*S*_y_* films were deposited during the ALD.

#### 3.1.2. Surface Morphology and Roughness

The Sn*_x_*S*_y_* films were strongly adhered to all of the substrate surfaces at deposition temperatures of, or lower than, 140 °C. These films on the glass were pale yellow. The color changed with the increase in the deposition temperature, as shown in Figure S1 (Supporting information). At 180 °C, the adherence of the Sn*_x_*S*_y_* films was low, and the color turned to black gray. All of the Sn*_x_*S*_y_* films seemingly remained smooth, shiny, and pinhole-free.

**Figure 2:**
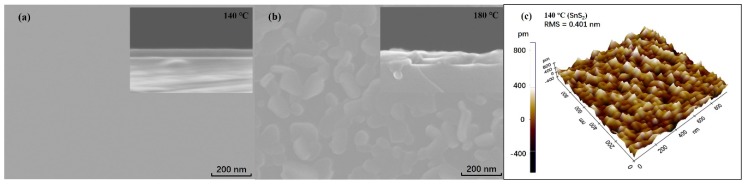
SEM micrographs of the Sn*_x_*S*_y_* films deposited at (**a**) 140 °C and (**b**) 180 °C on the Si wafer. The insets show the cross sections of the Sn*_x_*S*_y_* films. (**c**) Atomic force microscopy (AFM) micrograph of the surface of the Sn*_x_*S*_y_* film grown at 140 °C on the Si wafer. The root mean square roughness is 0.401 nm.

The microscale surface morphologies of the Sn*_x_*S*_y_* films deposited on the silicon wafer were observed using FESEM. As shown in Figure 2a, the Sn*_x_*S*_y_* film grown at 140 °C was very smooth and uniform (inset). Its surface and cross-section morphologies were similar as the fabrication temperature was lower. However, at 180 °C, an irregular distribution of flake grains was observed on the surface, and the film’s continuous but fluctuant cross-section exhibited a slightly nonuniform thickness, as shown in Figure 2b. Figure 2c shows an AFM image and surface roughness measurement results of the Sn*_x_*S*_y_* film grown at 140 °C on the silicon wafer. The root mean square (RMS) roughness is 0.401 nm, which indicates that the film is very flat.

#### 3.1.3. Crystal Structure and Phase Analysis

**Figure 3:**
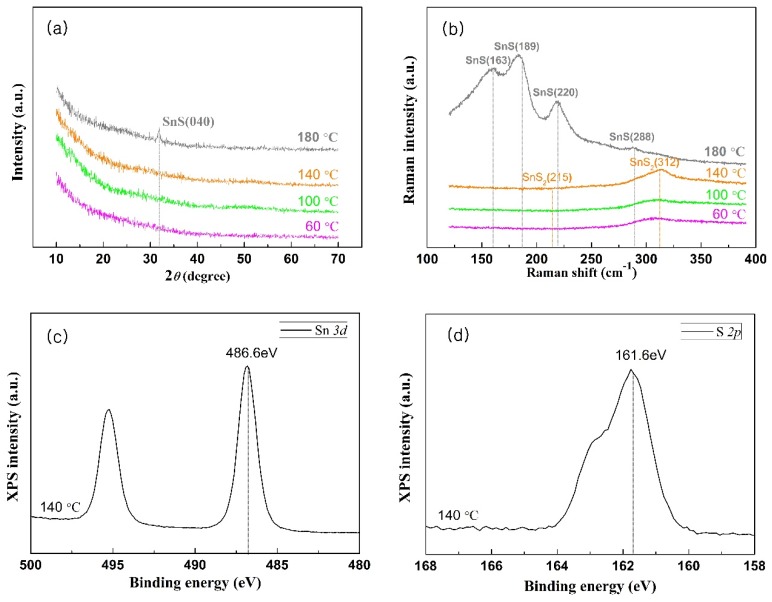
(**a**) XRD patterns of the Sn*_x_*S*_y_* films deposited at 60, 100, 140, and 180 °C on the glass substrate. The crystal structures of the Sn*_x_*S*_y_* films below 140 °C were amorphous, while at 180 °C they transformed into orthorhombic SnS with the preferred crystal orientation along the (040) plane. (**b**) Raman spectra of the Sn*_x_*S*_y_* films grown at 60, 100, 140, and 180 °C on the glass substrate. The Sn*_x_*S*_y_* films consisted of SnS_2_ at 60, 100, and 140 °C, while at 180 °C they transformed into SnS. X-ray photoelectron spectroscopy (XPS) of (**c**) Sn 3*d* and (**d**) S 2*p* peaks of the SnS_2_ film grown at 140 °C on the silicon wafer.

The XRD patterns showed that the crystal structures of the Sn*_x_*S*_y_* films transformed from the amorphous into the SnS orthorhombic structure when the deposition temperature was increased to 180 °C. As shown in Figure 3a, no peak was observed at 60, 100, and 140 °C, which indicates that the Sn*_x_*S*_y_* films were amorphous [24]. At 180 °C, an intense peak was observed at 2*θ* = 31.86°, which reflects the preferential orientation of the SnS film along the (040) plane, according to the Joint Committee on Powder Diffraction Standards (JCPDS) data (No. 39-0354) [31].

The Raman spectra indicated that when the deposition temperature was equal to or lower than 140 °C, the Sn*_x_*S*_y_* films consisted of SnS_2_, while at 180 °C, they transformed into SnS. As shown in Figure 3b, at the temperatures of 60, 100, and 140 °C, an obvious peak could be observed at 311.5 cm^−1^, while at 180 °C, peaks were observed at 161.6, 185.4, and 218.6 cm^−1^. These results are similar to those in previous studies. The Raman spectra have SnS_2_ bands at 312 and 215 cm^−1^ and SnS bands at 288, 220, 189, and 163 cm^−1^, as shown in Figure 3b [32,33].

Figure 3c,d shows the XPS Sn 3*d* and S 2*p* peaks of the Sn*_x_*S*_y_* film grown at 140 °C, respectively. The binding energy was calibrated using the C 1*s* peak (284.5 eV). The XPS Sn 3*d*_5/2_ and S 2*p*_3/2_ peaks are observed at 486.76 and 161.76 eV, respectively. These values are consistent with those of a previously reported SnS_2_ (486.6 and 161.6 eV, as shown in Figure 3c,d, respectively) [24].

**Figure 4:**
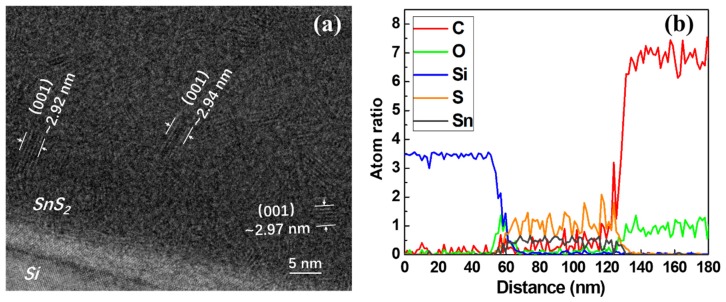
HRTEM image (**a**) and TEM-EDX line scanning profile (**b**) of the Sn*_x_*S*_y_* films deposited at 140 °C on the Si wafer.

As shown in Figure 4a, the high-resolution transmission electron microscopy image indicated that ALD SnS_2_ film deposited at 140 °C had a 2D crystalline structure in an amorphous phase matrix. There were many laminar structures with irregular distribution inside the film. By measuring the distance of the five layers, it had a lattice spacing of 0.589 Å, corresponding to the (001) interplanar distance of hexagonal SnS_2_ [22]. However, the ordered arrangement was irregularly distributed within a short distance of a few nanometers. Both Sn and S atoms exhibited disordered amorphous structures in the 50 nm thick film and the other two dimensions. Besides, Figure 4b showed that the average ratio of Sn to S atoms was ~1:2, but its fluctuation was very large, indicating that the distribution of tin and sulfur atoms was not very orderly.

In this study, the XRD, Raman spectroscopy, and XPS analyses show that the Sn*_x_*S*_y_* films consisted of amorphous SnS_2_ when the deposition temperature was equal to or lower than 140 °C. This result is consistent with HRTEM analysis. At 180 °C, the Sn*_x_*S*_y_* films transformed into orthorhombic SnS films with the preferred crystal orientation along the (040) plane. This observation can be explained by the thin film growth mechanism. The thin film growth mechanism is determined by the adsorption process on the substrate supplied by the deposition temperature [24,34,35]. The ALD growth of Sn*_x_*S*_y_* films as a function of temperature in our work was similar to previous study [24].

### 3.2. Optimization of the Optical Properties

**Figure 5:**
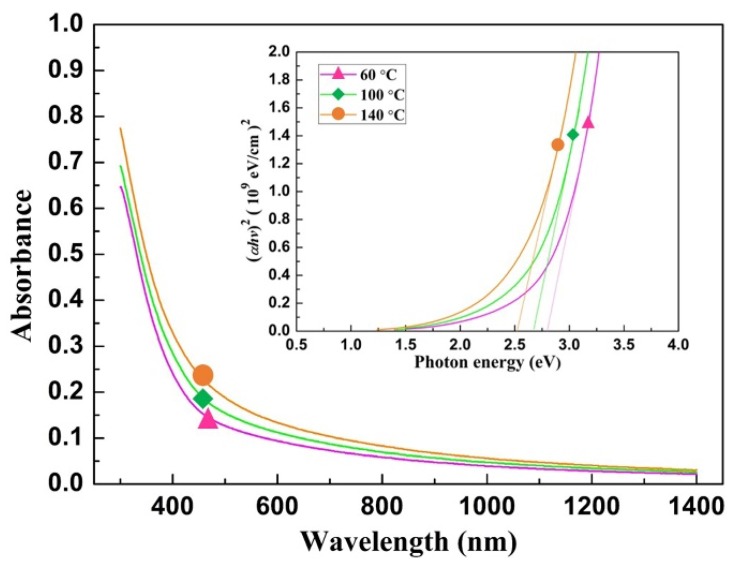
Absorbances and optical band gaps (inset) of the amorphous SnS_2_ films fabricated at different temperatures. The absorbance increased with the growth temperature, and the optical band gaps of the amorphous SnS_2_ were 2.80, 2.68, and 2.52 eV at 60, 100, and 140 °C, respectively.

The optical properties of the amorphous SnS_2_ films grown at different temperatures were measured in the UV–Vis range of 300 to 1400 nm. As shown in Figure 5, the absorbance of the amorphous SnS_2_ increases with the growth temperature. The optical band gap (*E*_g_) was obtained by measuring the transmittance and using equations *α* = [ln(1⁄*T*)]/*t* and *αE* = *A*(*E* − *E_g_* − *E*_p_)*^n^*, where *α* is the absorption coefficient, *T* is the transmittance, *t* is the film thickness, *E* (*hν*) is the photon energy, *A* is a constant, *E*_g_ is the optical band gap, and *E*_p_ is the phonon energy. For direct transitions (*E*_p_ = 0), *n* is equal to 1/2 for the allowed transition and 3/2 for the forbidden transition. For indirect transitions, *n* = 2 for the allowed transition and *n* = 3 for the forbidden transition [24,25,35,36,37]. In this study, the values of *α* of all of the films obeyed the above equation with *n* = 1/2, which indicates that all of the optical transitions between the valence and conduction bands were direct allowed transitions. The optical band gap of the amorphous SnS_2_ film was estimated by determining the intercept of the linear extrapolation with the photon energy for the absorption onset of the (*αhν*)^2^ curve. Figure 5 (inset) shows (*αhν*)^2^ as a function of *hν* for each temperature; the results are similar to previous study, which proved by using the ALD with varying deposition temperature to adjust optical band gaps of the Sn*_x_*S*_y_* films [24]. The summaries of crystal structure and optical band gap for each deposition temperature are shown in Table 1.

### 3.3. Photoelectrochemical Performances and Electrochemical Impedance Spectroscopy

#### 3.3.1. Photoelectrochemical Performances

**Figure 6:**
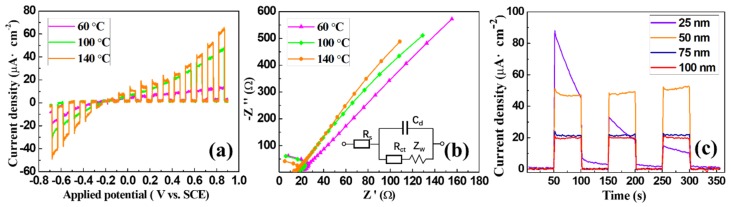
(**a**) Linear sweep voltammetry plots of the 50-nm-thick amorphous SnS_2_ films deposited at 60, 100, and 140 °C. (**b**) Nyquist plots and equivalent circuit of the 50-nm-thick amorphous SnS_2_ films deposited at 60, 100, and 140 °C. (**c**) Photocurrent response stabilities of the 25-, 50-, 75-, and 100-nm-thick amorphous SnS_2_ films fabricated at 140 °C. The stabilities of the photocurrent light-on–off responses were evaluated at 0.8 V (vs. SCE) during 360 s.

Linear sweep voltammetry plots of the 50-nm-thick amorphous SnS_2_ films grown at 60, 100, and 140 °C are shown in Figure 6a. Almost no photocurrent response was observed under dark conditions. However, under illumination, the SnS_2_ films very quickly responded to light. The photocurrent response increased with the applied voltage. As expected, the photocurrent response was the highest at 140 °C, as the lower optical band gap was beneficial to absorb and utilize more light energy. This result was three times higher than the photocurrent response of the SnS_2_ film grown at 60 °C. The SnS_2_ film grown at 140 °C exhibited high light–current density responses of −35.1 and 51.5 µA/cm^2^ at −0.6 and 0.8 V (vs. SCE), respectively. The light–current density responses were −12.4 and −20.2 µA/cm^2^ (−0.6 V vs. SCE) and 13.3 and 42.5 µA/cm^2^ (0.8 V vs. SCE) at 60 and 100 °C, respectively. This indicated that the ALD amorphous SnS_2_ films could be applied to hydrogen and oxygen evolution reactions, and the PEC performances of ALD amorphous SnS_2_ films could be optimized by varying the deposition temperature [38]. The amorphous SnS_2_ films have obvious photocurrent response to a negative voltage, but the photocurrent stability is very low.

#### 3.3.2. Electrochemical Impedance Spectroscopy

In the Nyquist plots of the SnS_2_ films, the charge transfer control was mainly reflected in the high-frequency area, while the diffusion control was mainly reflected in the low-frequency area. In the typical equivalent circuit of the electrolytic cell, the electrochemical polarization and concentration difference exist simultaneously, as shown in Figure 6b, where *R*_s_ is the solution resistance, *C*_d_ is the electric double-layer capacitance, *R*_ct_ is the charge transfer resistance, and *Z*_w_ is the Warburg impedance [39]. Based on sequential self-limiting reactions, ALD amorphous SnS_2_ film is excellently uniform and close contact with the substrate over large areas, leading to very small impedance. Moreover, the SnS_2_ film fabricated at a higher temperature not only absorbs more light energy but also has a smaller impedance, so that the photocurrent density is higher.

#### 3.3.3. Photocurrent Response Stabilities

The stabilities of the photocurrent responses of the films (fabricated at 140 °C) with thicknesses of 25, 50, 75, and 100 nm were evaluated at a voltage of 0.8 V for 360 s. The photocurrent response was initially stable when the film thickness was larger than 50 nm, as shown in Figure 6c. Although the photocurrent density of the film with 25 nm was very high at the moment of illumination (up to 88.0 µA/cm^2^), it rapidly decreased. When the thickness was larger than 50 nm, the photocurrent density became stable and decreased as the thickness increase. These results are expected; when the thickness of SnS_2_ film was 25 nm, photogenerated electrons were quickly transported to the electrode. However, the film was very thin so that the photocorrosion rapidly destroyed the double-layer structure. Films thicker than 50 nm could effectively stabilize the photocurrent by reducing the photocorrosion effect. However, the recombination of carrier and hole of the amorphous SnS_2_ film hindered the effective transmission of the photocurrent, which led to the decrease and stabilization of the photocurrent as the thickness increase. The photocurrent densities were 24.2 and 21.2 µA/cm^2^ at 75 and 100 nm, respectively. This is consistent with previous studies, which demonstrated that the nonmonotonous behavior of the photocurrent depends on the film thickness. It reaches its maximum under a certain thickness and then tends to stabilize [40,41,42].

In general, an excellent photocatalytic semiconducting material should have a high photoabsorption performance, high separation and transmission rates of photogenerated electrons and holes, and good stability [8]. The optimization of the optical band gap and thickness showed that the 50-nm-thick amorphous film deposited at 140 °C exhibited the best performances. The photocurrent density is higher than those of most crystalline SnS_2_ films. Furthermore, the measurement results of photoconversion efficiency [43,44,45,46] and the IPCE curve [22,47] of the 50-nm-thick SnS_2_ film deposited at 140 °C were shown in Figure S2. The maximum photoconversion efficiency (0.014%) was identified at ~0.553 V of bias (vs. SCE), and the highest IPCE value was up to 2.17% at 390 nm (0 V vs. RHE). Table 2 shows the PEC performances of SnS_2_ films reported in recent years. The photoelectron separation mechanism of these SnS_2_ films is discussed in Section 3.4.

### 3.4. Mechanisms of Photoelectron Separation and Photoelectrochemical Water Splitting

#### 3.4.1. Mechanism of Photoelectron Separation

**Figure 7:**
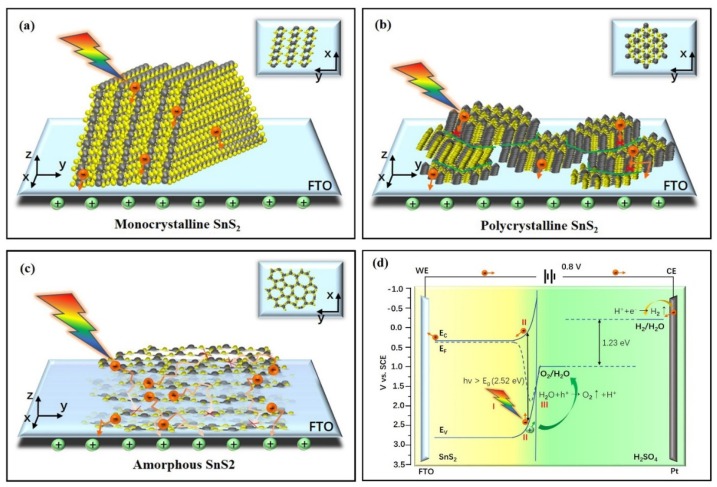
(**a**–**c**) Schematics demonstrating the efficient photon capture and charge transport from SnS_2_ to the FTO electrode: (**a**) monocrystalline, (**b**) polycrystalline, and (**c**) amorphous SnS_2_. The gray spheres represent tin atoms, while the yellow spheres represent sulfur atoms. (**d**) Schematic of SnS_2_ used in PEC water splitting. The main processes include (I) light energy absorption, (II) photoelectron–hole pair separation and transport, and (III) surface redox reaction.

It is well known that the basic characteristics of crystalline materials are the periodic arrangements of their constituent atoms, which could lead to anisotropic physical and chemical properties. However, for amorphous materials, the arrangement of atoms is not regular, and thus they are isotropic. Sun et al. [47] reported that freestanding SnS_2_ single-layers (three atomic layers’ thickness) exhibited a quite high photocurrent density of 2.75 mA/cm^2^ at 1.0 V vs. Ag/AgCl, more than 70 times higher than that of bulk SnS_2_. This is mainly attributed to the very large specific surface area, high percentage of disordered surface atoms, significantly better grain boundary connectivity, and intimate contact with the substrate. In addition, Guangbo Liu et al. [22] showed that vertically aligned 2D SnS_2_ NSs with full coverage on FTO (SnS_2_⊥FTO) exhibited a high photocurrent density of 1.73 mA/cm^2^ at 1.4 V vs. RHE, which is significantly higher than that of their parallel counterpart photoelectrode (SnS_2_//FTO). A schematic of the photoelectron transfer to the electrode is shown in Figure 7a. The good results can be attributed mainly to the SnS_2_ vertical growth and close contact with the substrate. The results indicated that the single-crystal structure is very favorable for the rapid separation and transfer of photoelectrons, as there are few obstacles to the movement of electrons within the crystal and the tight bonding with the substrate can reduce the resistance. However, it is usually very difficult to fabricate SnS_2_ in large areas with an ultrathin single-crystal structure or to control the growth direction of all of the crystalline SnS_2_ regions.

Jing et al. [12] studied PEC properties of SnS_2_ nanoparticles, SnS_2_ NSs, and 3D flower-like SnS_2_, with photocurrent densities of 13, 10, and 7.5 µA/cm^2^ at 1.0 V (vs. Ag/AgCl), respectively. SEM images showed that the SnS_2_ films were formed by random superposition of many small particles, NSs, or flower-like nanostructures. The highest photocurrent density of the SnS_2_ nanoparticles is attributed mainly to the best contact surface, highest interaction force, and preferred growth of crystal planes, compared with the other two materials. Monodisperse SnS_2_ NSs have been studied by Yu et al. [8], while Cheng et al. [9] studied vertical SnS_2_ NSs, whose photocurrent densities were 11.7 and 16.6 µA/cm^2^ at 0.8 and 0 V (vs. SCE), respectively. Their SEM morphologies are similar to those of the SnS_2_ NSs and 3D flower-like SnS_2_ studied by Jing, but the coverage seems to be better. A schematic of the photogenerated electron transfer from the NS to the electrode is shown in Figure 7b [8,12]. The photocurrent densities in these studies are significantly lower than that of the above monocrystalline SnS_2_. This can be attributed mainly to the superposition of many crystal particles, which lead to a large number of grain boundaries, as well as to the decrease in the tightness with the substrate. Both of these factors significantly affect the separation and transmission of photogenerated electrons.

A schematic of the photogenerated electron transfer from amorphous SnS_2_ to the electrode is shown in Figure 7c. It is well known that the arrangement of atoms is not regular in amorphous materials. The ALD amorphous SnS_2_ film is closely connected with the substrate, without any influence of grain boundaries. These facilitate charge carriers transfer between the film and conductive substrate [29]. However, the photogenerated electrons are also easy to recombine inside the film. Hence, as the thickness decreases, more photoelectrons move to the electrode before recombining. Considering the above-mentioned effects of light absorption and photocorrosion, the optimal stable photocurrent density was acquired at 50 nm deposited at 140 °C. This value is better than that of the above polycrystalline SnS_2_. It is worth noting that the working electrode structure, solution, and illumination conditions are different in each experiment. Therefore, we cannot directly accurately compare the photocurrent density values obtained from the different experimental systems. However, the above experiments confirmed that the crystal structure, SnS_2_ morphology, and state of bonding with the substrate have large impacts on the photocurrent response. In addition, in the experiment in this study, the amorphous films prepared by the ALD not only avoid the influence of defects such as grain boundaries, but also contribute to the improvement in photocurrent density by the smooth film tightly bound to the substrate. Furthermore, the photocurrent density was threefold increased by adjusting the optical band gap and thickness of the film. Therefore, it was reasonable that the 50-nm-thick amorphous SnS_2_ film grown at 140 °C exhibited the higher photocurrent density.

#### 3.4.2. Mechanism of Photoelectrochemical Water Splitting

Based on the above discussion and experimental results, the effect of SnS_2_ in PEC water splitting can be illustrated. As shown in Figure 7d, the complete PEC water splitting reaction involves three main processes. The first process is light absorption by the SnS_2_ photoelectrode. A photogenerated electron–hole pair is created as SnS_2_ absorbs a photon with an energy larger than its optical band gap. Therefore, the optical band gap is an important factor determining the degree of light energy absorption, which could be optimized in this study by adjusting the temperature of the SnS_2_ fabrication. The second process is the separation and transmission of photogenerated electron-hole pairs. These electrons are transported to the FTO electrode after the excitation to the conduction band, while the holes are in the valence band. In this process, the effective separation and transmission are necessary as electron–hole pairs can recombine at the surface or in the bulk. Therefore, the SnS_2_ film thickness is optimized. The third process is the surface reaction, where holes can cause an oxygen evolution reaction in the solution [38,48].

## 4. Conclusions

SnS_2_ amorphous films were fabricated by ALD and applied for the first time to PEC experiments. The 50-nm-thick SnS_2_ film fabricated at 140 °C had the maximum photocurrent density of 51.5 µA/cm^2^ at 0.8 V (vs. SCE), which is better than those of most polycrystalline SnS_2_ films. This demonstrated that the amorphous SnS_2_ films could be used for PEC water splitting. Furthermore, by comparing the mechanism of the photogenerated electron transfer from the amorphous, monocrystalline, and polycrystalline SnS_2_ to the electrode, the reason for high photocurrent density of thin amorphous SnS_2_ film was explained theoretically. Last, the schematic of the PEC water splitting mechanism was presented.

## Figures and Tables

**Table 1:** The summaries of crystal structure and optical band gap for each deposition temperature.

Deposition Temperature (°C)	Crystal Structure	Optical Band Gap (eV)
60	SnS_2_ Amorphous	2.80
100	SnS_2_ Amorphous	2.68
140	SnS_2_ Amorphous	2.52
180	SnS orthorhombic	1.78

**Table 2:** Comparison of the SnS_2_ photoelectrochemical (PEC) performances with those in other studies.

No.	Working Electrode Structure	Photocurrent Density (µA/cm^2^)	Reference Electrode (Applied Potential)	Solution	Irradiation	Reference (Year)
1	Glass/ITO/SnS_2_ (single-layer)	2750	Ag/AgCl (1.0 V)	0.5 M Na_2_SO_4_	300 W Xe lamp irradiation (*λ* > 420 nm)	47 (2012)
2	Glass/ITO/SnS_2_ (bulk)	<5				
3	Glass/ITO/SnS_2_ NSs (with PVP)	11.7	SCE (0.8 V)	0.5 M Na_2_SO_4_	300 W Xe lamp illumination (*λ* > 420 nm)	8 (2014)
4	Glass/ITO/SnS_2_ NSs (without PVP)	3.7				
5	Glass/ITO/SnS_2_ NSs (water and 0.5 g PVP)	2.3				
6	Glass/FTO/Ni/SnS_2_/C (FNSC)	38.6	SCE (0 V)	0.5 M Na_2_SO_4_	Xenon lamp, 100 mW cm^-2^ (*λ* > 420 nm)	9 (2016)
7	Glass/FTO/Ni/SnS_2_ (FNS)	19.8				
8	Glass/FTO/SnS_2_ (FS)	16.6				
9	CC⊥SnS_2_	1920	RHE (1.4 V)	0.5 M Na_2_SO_4_	AM 1.5 G 100 mW cm^−2^ solar light	22 (2017)
10	Glass/FTO⊥SnS_2_	1730				
11	Glass/FTO//SnS_2_	910				
12	Glass/Cr/Au/SnS_2_	−2	Ag/AgCl (−0.4 V)	0.25 M H_2_SO_4_	Simulated AM 1.5 sunlight (100 mW cm^−2^)	38 (2017)
13	Glass/Cr/Au/SnS_2_	195	Ag/AgCl (0.8 V)			
14	g-C_3_N_4_/SnS_2_ nanoparticle	13	Ag/AgCl (1.0 V)	0.5 M Na_2_S	300 W xenon lamp (*λ* > 400 nm)	12 (2018)
15	g-C_3_N_4_/SnS_2_ NS	10				
16	g-C_3_N_4_/3D flower-like SnS_2_	7.5				
17	Glass/FTO/SnS_2_ (amorphous)	−35.1	SCE (−0.6 V)	0.25 M H_2_SO_4_	Simulated AM 1.5 sunlight (100 mW cm^−2^)	This study
18	Glass/FTO/SnS_2_ (amorphous)	51.5	SCE (0.8 V)			

ITO: indium tin oxide; PVP: polyvinylpyrrolidone; CC: carbon cloth; 3D: three-dimensional.

## Supplementary Materials

The following are available online at https://www.mdpi.com/2079-4991/9/8/1083/s1. Figure S1: Photos of 50-nm-thick Sn*_x_*S*_y_* films which in the growth of the glass substrates at different deposition temperatures. Figure S2: Photoconversion efficiency (a) and IPCE curve (b) of the 50-nm-thick SnS_2_ film deposited at 140 °C.
