# Supplementary material for: Enhancement in Photoelectrochemical Performance of Optimized Amorphous SnS2 Thin Film Fabricated through Atomic Layer Deposition
Source: Nanomaterials (Basel). 2019 Jul 28;9(8):1083. doi: 10.3390/nano9081083 (PMC6723338; doi:10.3390/nano9081083)
Supplement: Supplementary file 1 [file nanomaterials-09-01083-s001.pdf]

nanomaterials

Supplementary materials

Enhancement in Photoelectrochemical Performance of Optimized Amorphous SnS_2 Thin Film Fabricated Through Atomic Layer Deposition

Weiguang Hu [1], Truong Thi Hien [2], Dojin Kim [2], Hyo Sik Chang [1,]*

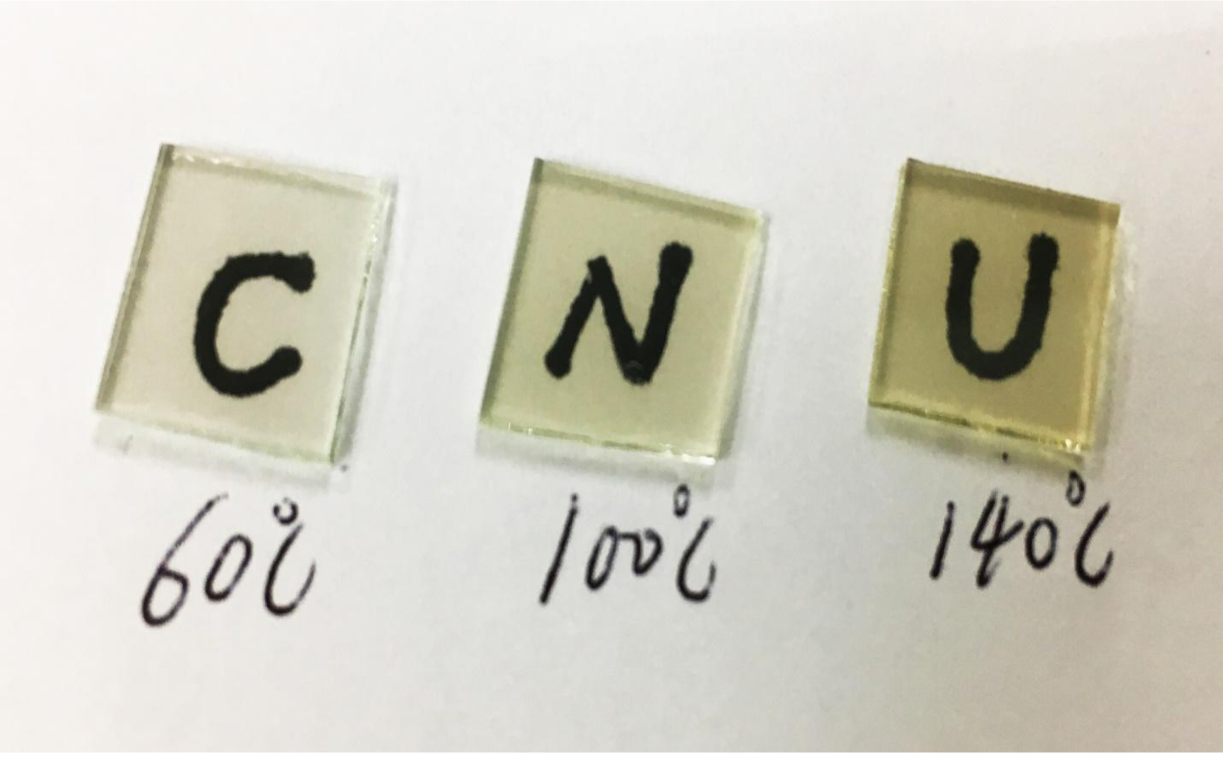

Figure S1. Photos of 50-nm-thick Sn_xS_y films which in the growth of the glass substrates at different deposition temperatures. The C, N, and U represent the deposition temperature of 60, 100, and 140 °C, respectively. All of them are pale yellow and the color deepens with the increase of deposition temperature.

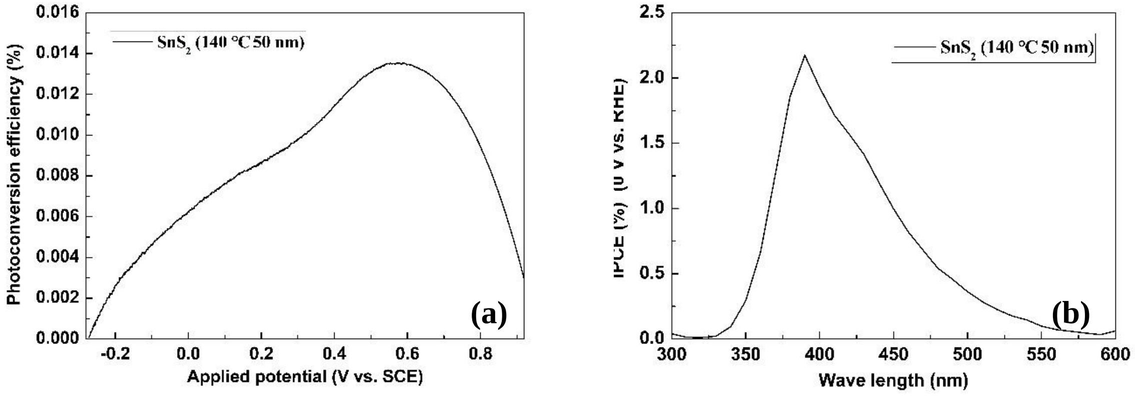

Figure S2. Photoconversion efficiency **(a)** and IPCE curve **(b)** of the 50-nm-thick SnS_2 film deposited at 140 °C. Photoconversion efficiency was measured as a function of applied potential (vs. SCE) under illumination. The maximum efficiency (0.014%) was identified at ~0.553 V of bias (vs. SCE). The highest IPCE value was up to 2.17% at 390 nm (0 V vs. RHE).

© 2019 by the authors. Submitted for possible open access publication under the terms and conditions of the Creative Commons Attribution (CC BY) license (http://creativecommons.org/licenses/by/4.0/).
